# Determination of Seebeck coefficient originating from phonon-drag effect using Si single crystals at different carrier densities

**DOI:** 10.1038/s41598-023-40685-6

**Published:** 2023-08-18

**Authors:** Masataka Hase, Daiki Tanisawa, Kaito Kohashi, Raichi Kamemura, Shugo Miyake, Masayuki Takashiri

**Affiliations:** 1 Department of Materials Science, Tokai University, 4–1–1 Kitakaname, Hiratsuka, Kanagawa 259–1292 Japan; 2 Department of Mechanical Engineering, Kobe City College of Technology, 8-3 Gakuenhigashi-Machi, Kobe, Hyogo 651–2194 Japan

**Keywords:** Energy science and technology, Materials science

## Abstract

The phonon-drag effect is useful for improving the thermoelectric performance, especially the Seebeck coefficient. Therefore, the phonon and electron transport properties of Si single crystals at different carrier densities were investigated, and the relationship between these properties and the phonon-drag effect was clarified. Phonon transport properties were determined using nanoindentation and spot-periodic heating radiation thermometry. The electron transport properties were determined based on the electrical conductivity of Si. The diffusive Seebeck coefficient derived from the electron transport properties was in good agreement with previous reports. However, the value of the phonon-drag Seebeck coefficient derived from the phonon transport properties is very low. This phenomenon suggests that phonons with a normal mean free path (MFP) do not contribute to the increase in the Seebeck coefficient; however, phonons with a long MFP and low frequency increase the Seebeck coefficient via the phonon-drag effect. Moreover, the phonon-drag effect was sufficiently pronounced even at 300 K and in the heavily doped region. These features are key in designing thermoelectric materials with enhanced performance derived from the phonon-drag effect.

## Introduction

From an energy harvesting perspective, phonon engineering is gaining significant attention due to the increasing demand for materials with controllable heat transfer properties^1–5^. Theoretical analysis is essential for this realization, and understanding the detailed mechanism of phonon transport is an effective strategy for material development. In recent years, many studies have analyzed phonon transport through simulations^6–8^, however, it is equally important to evaluate these results experimentally.

For some materials that utilize thermal energy, electron transport also affects the material’s performance. Particularly in thermoelectric conversion materials, the carrier density plays an important role in electron transport^9–11^. Thermoelectric materials generate thermoelectric power proportional to the Seebeck coefficient by the transfer of charge carriers owing to temperature differences. An ideal thermoelectric material exhibits both high electrical conductivity and low thermal conductivity; however, microscopically, the interaction between phonons and carriers strongly affects these conductivities.

A theoretical analysis of the phonon-drag effect was also carried out to develop materials with low thermal and high electrical conductivities^12,13^. In general, phonon-drag is a phenomenon often observed in conditions which facilitate long phonon mean free paths (MFP), such as low-temperature surroundings and high-purity materials. However, it has been reported that Si and Si-based alloys exhibit relatively high Seebeck coefficients originating from the phonon-drag effect even at room temperature^14–16^. Hence, to increase the thermoelectric performance, it is necessary to study phonon/electron transport properties at different carrier densities and their impact on the phonon-drag effect.

In this study, Si single crystals with different amounts of phosphorus doping were used because the crystals were perfectly oriented with no grain boundaries, and the carrier density could be easily varied. For the evaluation of phonon transport properties, we determined group velocities and phonon MFP of various materials using nanoindentation and thermal conductivity measurements^17,18^. The effect on the crystal orientation of the Si single crystals was investigated using the same technique^19^. Hence, this technique can be used to evaluate the phonon transport in Si single crystals at different carrier densities. Electron transport properties were obtained from the measured electrical conductivities of the Si single crystals. The relationship between carrier density and phonon/electron transport properties was investigated. Finally, the phonon-drag phenomenon was investigated based on phonon/electron transport properties. A novel finding of this study is that phonons with a normal mean free path (MFP) do not contribute to increasing the Seebeck coefficient, whereas phonons with a long MFP increase the Seebeck coefficient via the phonon-drag effect. In addition, the phonon-drag effect was sufficiently pronounced even at 300 K and in the heavily doped region. These outcomes are significant for designing thermoelectric materials with enhanced performance derived from the phonon-drag effect.

## Results

Table 1 lists the physical properties of the Si single crystals measured at 300 K. The carrier density was derived from the measured electrical conductivity using the method reported in the literature^20^. Notably, the carrier density was almost the same as the doping concentration at 300 K determined from the previous literature^21^. The carrier density of the undoped sample was determined to be 1.8 × 10^11^ cm^−3^. The intrinsic carrier density of Si at 300 K is 1.5 × 10^10^ cm^−3^, and thus, the carrier density of undoped Si in this study is reasonable^20^. The carrier density significantly increased to 7.0 × 10^18^ cm^−3^ with increasing electrical conductivity. The mobility *μ* is estimated from *μ* = *σ*/*qn*, where *σ*, *q*, and *n* are the electrical conductivity, elementary charge, and carrier density, respectively. The mobility decreased significantly with an increase in carrier density. This was due to an increase in the impurity scattering frequency of the electrons as the carrier density increased^22^.

**Table 1:** Electrical properties measured at 300 K and the carrier density of Si single crystals.

Sample	Crystal orientation	Dopant	Electrical conductivity *σ* [S/cm]	Doping concentration *n* [cm^−3^]	Carrier density *n* [cm^−3^]	Mobility *μ* [cm^2^/(V-s)]
Sample #1	[111]	Undoped	5.6 × 10^–5^	–	1.8 × 10^11^	2000
Sample #2	[111]	Phosphorus	5.2 × 10^–2^	2.3 × 10^14^	2.3 × 10^14^	1460
Sample #3	[111]	Phosphorus	1.3	6.6 × 10^15^	6.6 × 10^15^	1259
Sample #4	[111]	Phosphorus	1.7 × 10	1.6 × 10^17^	1.5 × 10^17^	683
Sample #5	[111]	Phosphorus	1.4 × 10^2^	7.0 × 10^18^	7.0 × 10^18^	126

**Figure 1:**
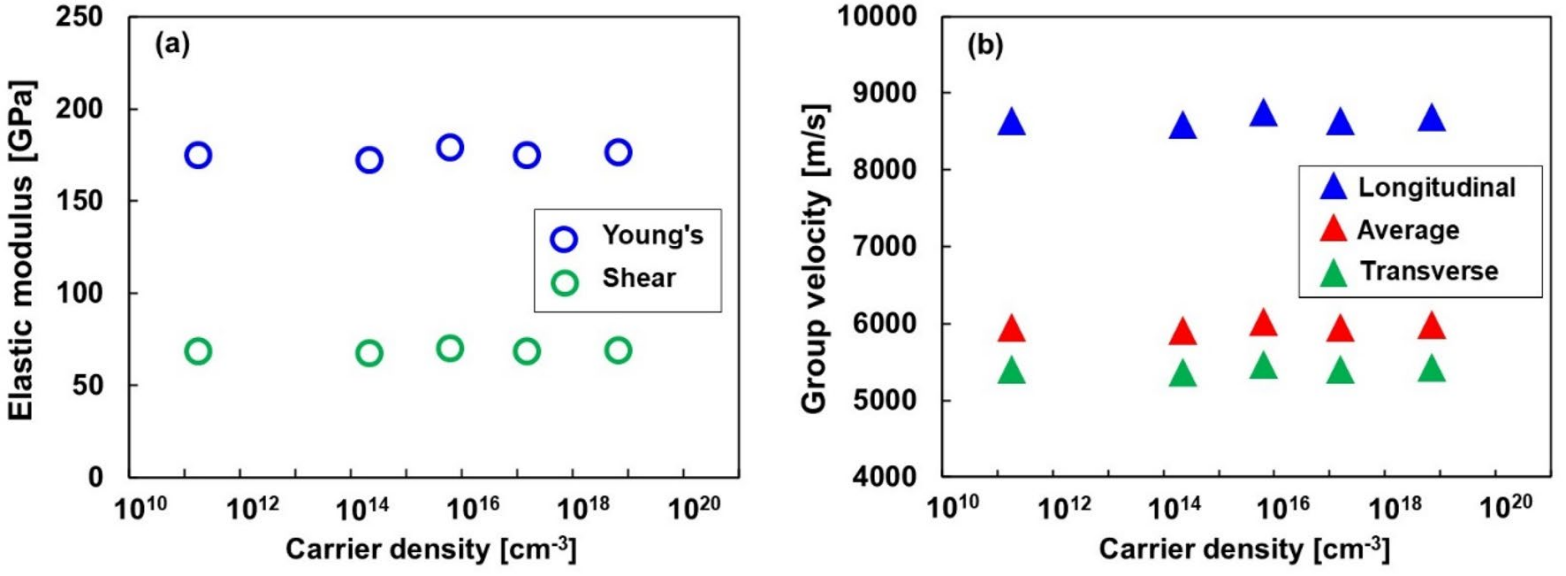
(**a**) Young's and shear moduli obtained by nanoindentation and (**b**) group velocity obtained from Young's and shear moduli.

By analyzing the load-depth curves obtained from nanoindentation, the elastic modulus and group velocity of the Si single crystals at different carrier densities were evaluated. The load-depth curves of the Si single crystals at different carrier densities are shown in the Supplementary Information (Fig. S1). The carrier density dependences of the Young's and shear moduli are shown in Fig. 1a. A Poisson's ratio of 0.28 was used for all carrier densities. Both Young's and shear moduli were independent of the carrier density and showed constant values of 175 and 68 GPa, respectively. Figure 1b shows the group velocity as a function of carrier density. The longitudinal group velocity *v*_*L*_ and transverse group velocity *v*_*T*_ were determined from the Young's modulus and shear modulus, respectively. The average group velocity *v*_ave_ is described as 3/*v*_ave_^3^ = 1/*v*_*L*_^3^ + 2/*v*_*T*_^3^. The group velocities were mostly independent of the carrier density because the doping concentration was significantly lower than the density of silicon (5.0 × 10^22^ cm^−3^). Therefore, the dopants did not affect the elastic modulus or group velocities; similar results have been reported by Hall^23^. The longitudinal, transverse, and average group velocities were 8667, 5416, and 5967 m s^−1^, respectively.

**Figure 2:**
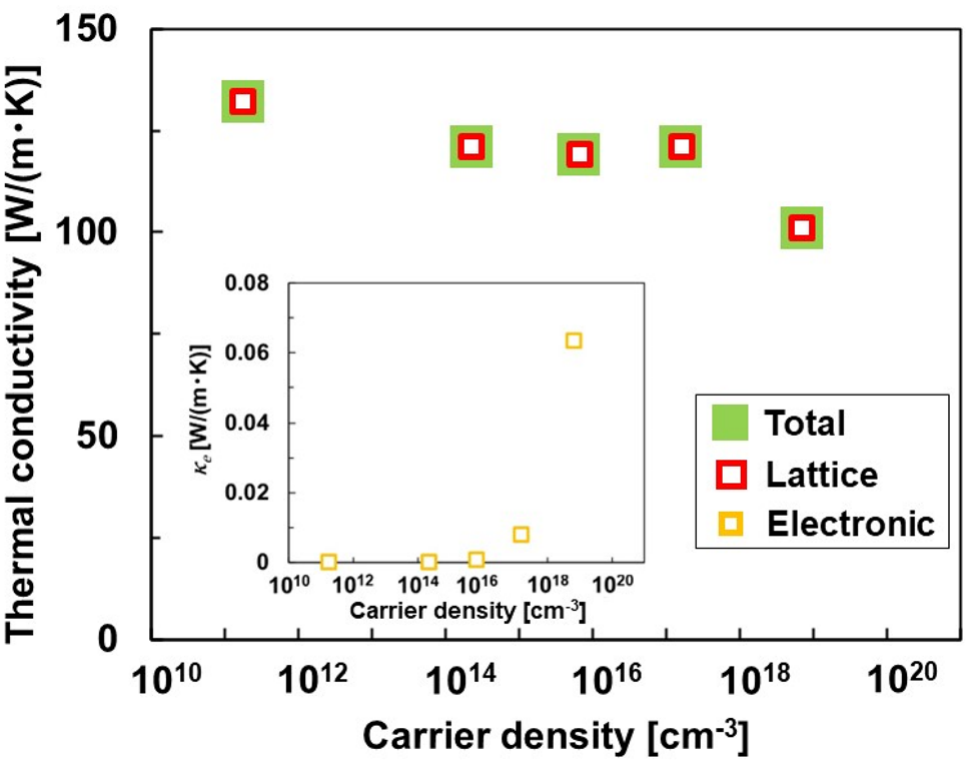
Thermal conductivities of Si single crystals at different carrier densities. The inset shows the electronic thermal conductivity.

Figure 2 shows the thermal conductivities of the Si single crystals at different carrier densities. The lattice thermal conductivity (*κ*_*l*_) was obtained by excluding the electronic thermal conductivity (*κ*_*e*_) from the measured total thermal conductivity (*κ*_total_). *κ*_*e*_ was calculated using the measured electrical conductivity and the Wiedemann–Franz law. Here, a Lorenz number of 1.5 × 10^–8^ W Ω K^−2^ for non-degenerate semiconductors was used with the Wiedemann–Franz law because the carrier density of all samples was less than 10^20^ cm^−3^^24,25^. The *κ*_total_ of undoped Si was measured as 132 W (m K)^−1^. The *κ*_total_ of Si showed a constant value of 120 W (m K)^−1^ for carrier density ranging from 2.3 × 10^14^ to 1.6 × 10^17^ cm^−3^. However, it decreased to 101 W (m K)^−1^ when the carrier density was 7.0 × 10^18^ cm^−3^. This phenomenon occurred because the effects of impurity scattering became prominent when the doping concentration was higher than 10^17^ cm^−3^^26,27^. *κ*_*l*_ is mostly equal to the corresponding *κ*_total_ because of negligible *κ*_*e*_, as shown in the inset of Fig. 2, even though *κ*_*e*_ increases with increasing carrier density.

**Figure 3:**
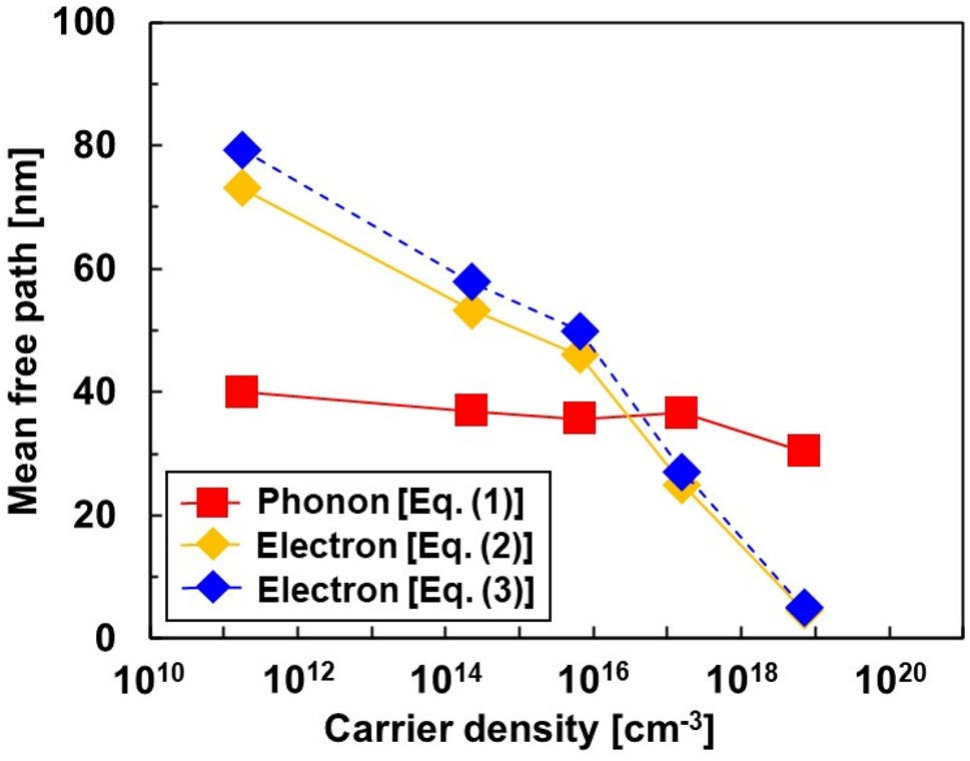
Phonon and electron MFPs as a function of carrier density.

Figure 3 shows the phonon and electron MFPs of the Si single crystals at different carrier densities. The Phonon MFP *Λ*_*p*_ was determined using Eq. (1), based on the phonon gas model:

1 $${\Lambda }_{p}=\frac{3{\kappa }_{l}}{Cv_{ave}},$$

where *C* is the specific heat and *v*_*ave*_ is the average group velocity. The electron MFP *λ*_*e*_ was determined using Eq. (2):

2 $${\lambda }_{e}={v}_{e}{\tau }_{e},$$

where *v*_*e*_ is the velocity of the electrons, and *τ*_*e*_ is the relaxation time. In non-degenerate Si, *v*_*e*_ corresponds to the thermal velocity *v*_*th*_, expressed as *v*_*th*_^2^ = 3*k*_*B*_*T*/m^*^, where *k*_*B*_ is the Boltzmann constant, *T* is the absolute temperature, and m^*^ is the effective mass^28^. An effective mass of m^*^ = *m*_0_/3.3 was used in this study^29^, where *m*_0_ is the free electron mass, and *τ*_e_ is determined by the equation *τ*_e_ = μm^*^/*q*, where the mobility *μ* is listed in Table 1. The electron MFP under the condition that acoustic phonon scattering is dominant is expressed by Eq. (3)^30^.

3 $${\lambda }_{e}=\frac{3\mu {\left(2\pi {m}^{*}{k}_{B}T\right)}^\frac{1}{2}}{4q}$$

For undoped Si, the phonon MFP was obtained at 40 nm. As undoped Si is almost free of carriers and grain boundaries, the main phonon scattering is Umklapp scattering. When the carrier density was in the range of 2.3 × 10^14^–1.6 × 10^17^ cm^−3^, the phonon MFP showed a constant value of 36 nm, which was 10% lower than that of undoped Si. As the doping concentration is almost proportional to the carrier density, a phonon impurity scattering effect occurs, and the phonon MFP decreases as the doping concentration increases. However, at these carrier densities, the fraction of phonon and impurity scattering was low compared with the Umklapp scattering. When the carrier density was further increased, the phonon MFP decreased to 30 nm, which is 25% lower than that of undoped Si. Therefore, the effect of phonon impurity scattering was more pronounced at the carrier density on the order of 10^17^ cm^−3^.

In contrast, based on Eq. (2), the electron MFP of undoped Si is 73 nm, which is larger than that of the phonon MFP. The electron MFP decreased significantly with increase in carrier density. At carrier densities in the order of 10^16^ cm^−3^, the electron MFP became lower than the phonon MFP. Furthermore, at a carrier density of 7.0 × 10^18^ cm^−3^, the electron MFP dropped to 4.6 nm. On comparing the electron MFP obtained from Eqs. (2) and (3), both values show good agreement and are almost identical in the high-carrier-density region. Overall, the electron MFP is more dependent on the carrier density than the phonon MFP because electrons are charged particles and are consequently affected by Coulomb potentials derived from impurities, whereas phonons are lattice vibrations and are not influenced by Coulomb potentials.

**Figure 4:**
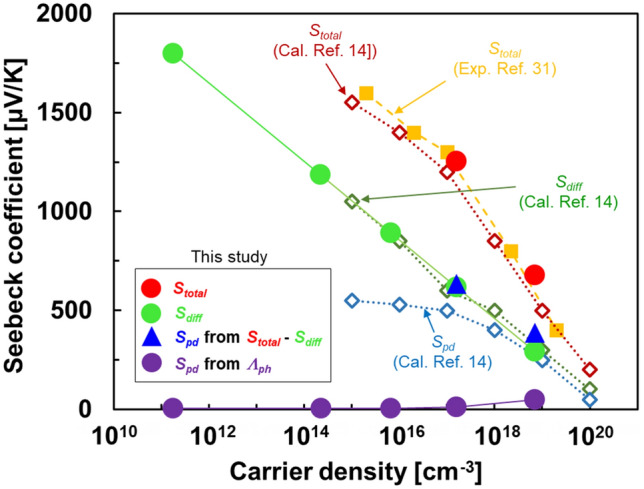
Variation of Seebeck coefficient at 300 K as a function of carrier density.

Figure 4 shows the Seebeck coefficients of the Si single crystals at different carrier densities at 300 K. The total Seebeck coefficient (*S*_total_) obtained in this study was measured experimentally. The diffusive Seebeck coefficient (*S*_diff_) was estimated from Eq. (4), which is mainly based on the carrier density:

4 $${S}_{\mathrm{diff}}=-\frac{{k}_{B}}{q}\left[\mathrm{ln}\left(\frac{n}{{n}_{0}}\right)-\frac{\Delta \varepsilon }{{k}_{B}T}\right],$$

where Δ*ε* is the energy of the electron for the conduction band edge (*ε*_*c*_) given by Δ*ε* = 2*k*_*B*_*T*, and* n*_0_ is the effective density of states of the conduction band expressed by Eq. (5):

5 $${n}_{0}=2{\left(\frac{2\pi {m}_{d}{k}_{B}T}{{h}^{2}}\right)}^\frac{3}{2},$$

where *m*_*d*_ is the effective mass of the density of states which is obtained as *m*_*d*_ = 1.08*m*_*e*_. We evaluated the Seebeck coefficient derived from phonon-drag (*S*_pd_) in two approaches. The first uses Eq. (6), which is mainly based on the experimental values of the group velocity, phonon MFP, and electrical conductivity:

6 $${S}_{\mathrm{pd}}=\frac{\beta v_{ave}nq{\Lambda }_{\mathrm{P}}}{\sigma T},$$

where *β* is a parameter that determines the relative strength of the electron–phonon interaction and has a range of 0 < *β* < 1, and we used *β* = 1 in this study^28^. The second is to subtract *S*_diff_ from *S*_total_ (*S*_pd_ = *S*_total_ − *S*_diff_) because *S*_total_ consists of *S*_diff_ and *S*_pd_. The total Seebeck coefficient varied from − 1252 to − 679 μVK^−1^ when the carrier density increased from 1.6 × 10^17^ to 7.0 × 10^18^ cm^−3^. The total Seebeck coefficients in this study are in good agreement with the previous reports^14,31^. Notably, accurate Seebeck coefficients could not be measured at carrier densities less than 6.6 × 10^15^ cm^−3^ in our measurement system. The undoped Si single crystal exhibited a diffusive Seebeck coefficient of − 1800 μV K^−1^, and its absolute value decreased with increase in carrier density. These phenomena are in good agreement with those reported previously^14^.

The values of *S*_pd_ estimated from *S*_total_ − *S*_diff_ were − 633 and − 402 μV K^−1^ at carrier densities of 1.6 × 10^17^ and 7.0 × 10^18^ cm^−3^, respectively. These are approximately half the values of *S*_total_ at the corresponding carrier densities and similar values are obtained using calculations from literature^14^, indicating that the phonon-drag effect is evident near 300 K. In contrast, *S*_pd_ estimated from Eq. (6) was almost zero when the carrier density was less than 1.6 × 10^17^ cm^−3^. Even though the *S*_pd_ from Eq. (6) negatively increased to − 48 μVK^−1^ at a carrier density of 7.0 × 10^18^ cm^−3^, the value was approximately ten times lower than *S*_pd_ estimated from *S*_total_ − *S*_diff_. This difference occurred because the phonon-drag effect is due to the contribution of lower-frequency (longer-wavelength) phonons interacting with electrons^16^.

**Figure 5:**
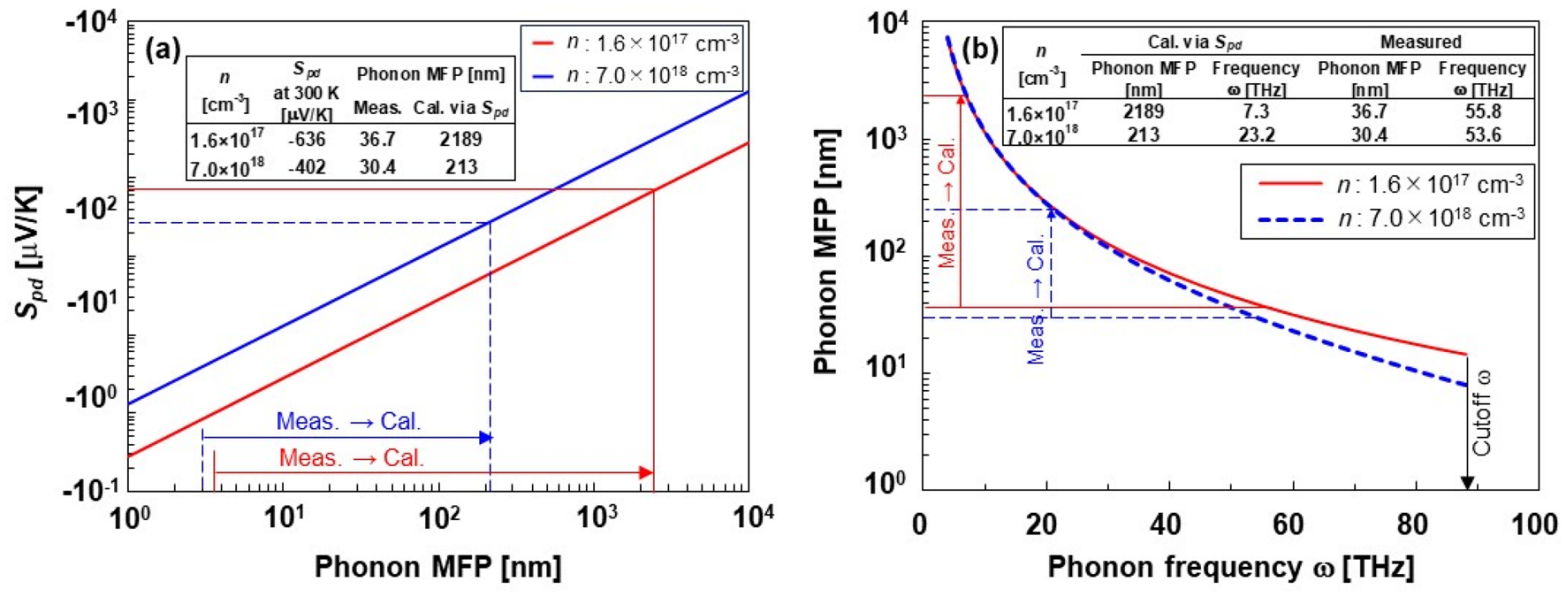
(**a**) Relationship between phonon-drag Seebeck coefficients at 300 K and phonon MFP and (**b**) relationship between phonon MFP and frequency.

Consequently, the phonon MFP contributing to the phonon-drag effect for each carrier density was calculated as shown in Fig. 5a. In this calculation, the phonon MFP was calculated by inserting the values of *S*_pd_ obtained from *S*_total_ − *S*_diff_ into Eq. (6). Notably, the other parameters in Eq. (6) are the same as those in the abovementioned calculations. The calculated phonon MFP was 2189 nm at 1.6 × 10^17^ cm^−3^ and 213 nm at 7.0 × 10^18^ cm^−3^. These values were significantly larger than those of the phonon MFP, contributing to thermal conduction obtained from the measurements by a factor of 10–100. The contribution of the phonon MFP to the phonon-drag effect is more dependent on the carrier density, which corresponds to the calculations of Herring^32^. To further investigate the phonon-drag effect, the phonon frequencies were calculated at different carrier densities at 300 K using the models proposed by Slack and coworkers^33,34^. The phonon scattering mechanisms considered in this study include Umklapp scattering (*Λ*_*um*_) and impurity scattering (*Λ*_*imp*_), which are combined using Matthiessen's rule:

7 $${{\Lambda }_{p}}^{-1}\left(\omega , T\right)={{\Lambda }_{um}}^{-1}\left(\omega , T\right)+{{\Lambda }_{imp}}^{-1}\left(\omega \right).$$

For Umklapp scattering, the relaxation time *τ*_*U*_ is determined using the following semi-empirical equation:

8 $${{\tau }_{um}}^{-1}={A}_{um}{\omega }^{2}T.$$

Here, *A*_*um*_ is approximated from the relation:

9 $${A}_{um}\sim \frac{h{\gamma }^{2}}{2\pi m\theta {{v}_{ave}}^{2}}\mathrm{exp}\left(-\theta /3T\right),$$

where the Grüneisen parameter (*γ*) is assumed to be equal to 2, and *m* is the average mass of a single atom (*m* = 4.65 × 10^–26^ kg); *θ* is the Debye temperature (*θ* = 674 K). Therefore, *Λ*_*um*_ is calculated using the following equation:

10 $${\Lambda }_{um}={v}_{ave}{\tau }_{um}.$$

For impurity scattering, *Λ*_*imp*_ is calculated using the following equation:

11 $${\Lambda }_{imp}=\frac{{v}_{ave}}{B{\omega }^{4}}.$$

Here, the impurity scattering *B* is assumed to be linearly dependent on the impurity concentration:

12 $$B={B}_{i}{x}_{i},$$

where *x*_*i*_ is the concentration of impurity *i*. In this study, the impurity corresponds to the phosphorous dopant. The proportionality factor *Bi* = 8.62 × 10^–70^ s^3^ m^3^ was determined by fitting the experimental data presented in a previous report^26^. The relationship between the phonon MFP and frequency is shown in Fig. 5b. In the low-frequency range below 20 THz, the relationship between the phonon MFP and the frequency is independent of the carrier density. However, when the phonon frequency exceeded 20 THz, the phonon MFPs tended to be shorter at higher carrier densities in the heavily doped regions. The cutoff frequency was estimated to be 88.2 THz from the Debye temperature. At a lower carrier density of 1.6 × 10^17^ cm^−3^ in the lightly doped region, the measured and calculated phonon MFPs were 36.7 and 2189 nm, corresponding to phonon frequencies of 55.8 and 7.3 THz, respectively. On the other hand, at a higher carrier density of 6.0 × 10^18^ cm^−3^, the measured and calculated phonon MFPs were 30.4 and 213 nm, corresponding to phonon frequencies of 53.6 and 23.2 THz, respectively. Therefore, we conclude that phonons contributing to the phonon-drag effect have a longer MFP and lower frequencies, and that lower-frequency phonons contribute to the phonon-drag effect in the lightly doped region.

## Discussion

As shown in Figs. 3, 4 and 5, the phonon MFP contribution to the thermal conduction depends on the carrier density, but the effect of impurity scattering is more significant^35^. The contribution of phonons with longer MFPs and lower frequencies to the phonon-drag effect depends on the carrier density. These results suggest the possibility of decreasing the thermal conductivity while maintaining a high Seebeck coefficient with the phonon-drag effect by optimizing the carrier density.

**Figure 6:**
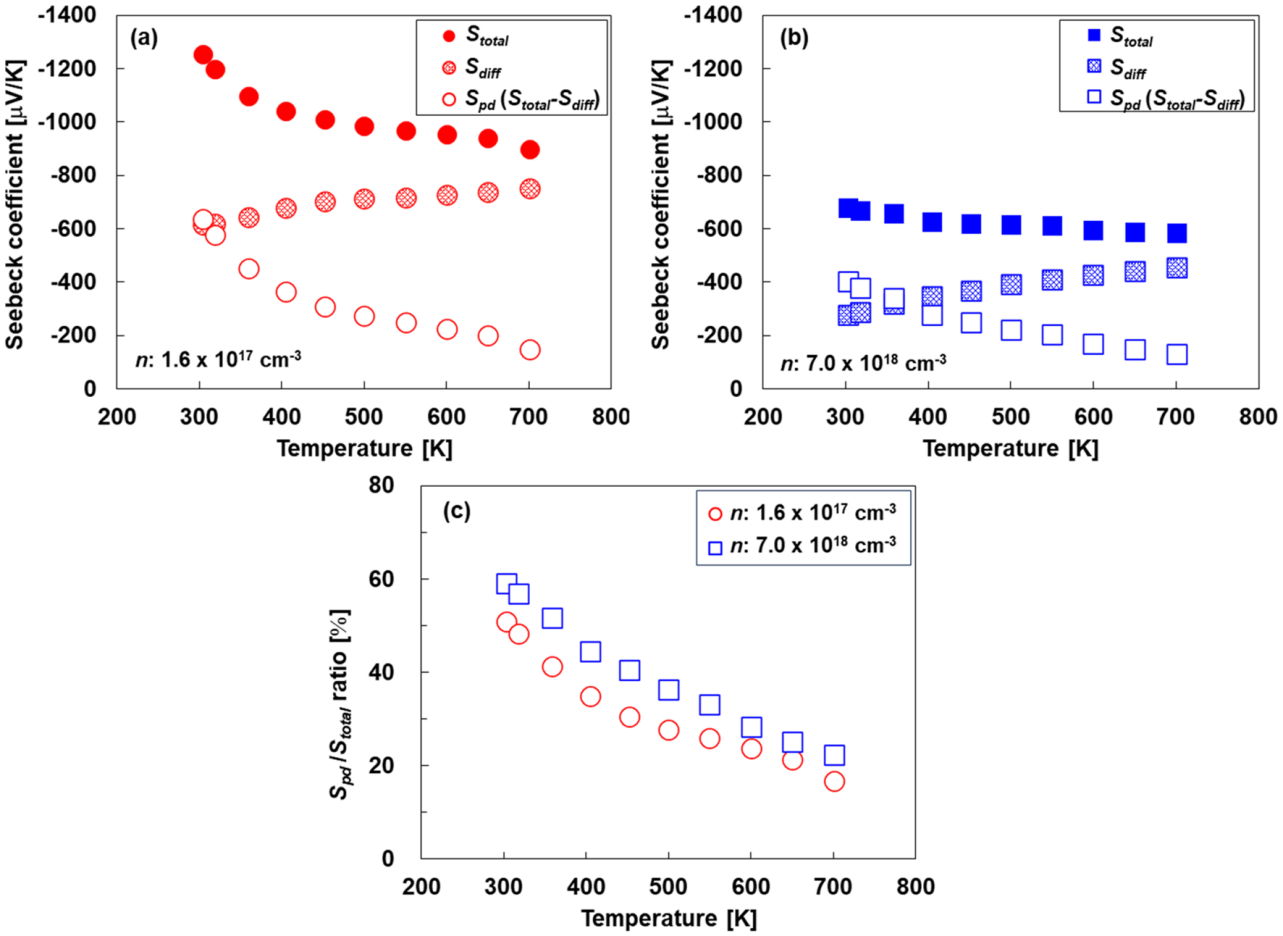
Temperature-dependence of Seebeck coefficient at (**a**) 1.6 × 10^17^ cm^−3^ and (**b**) 7.0 × 10^18^ cm^−3^, (**c**) contribution of phonon-drag effect to Seebeck coefficient; ratio of *S*_pd_ to *S*_total_ as a function of temperature.

To validate the proposals, the Seebeck coefficient (*S*_total_) was measured in the range of 300–700 K with different carrier densities. *S*_diff_ and *S*_pd_ (*S*_total_ − *S*_diff_) were calculated using the same methods presented in the “Results” section. In general, the phonon-drag effect is much stronger below room temperature^36,37^; however, herein, the Seebeck coefficient was investigated in the temperature range of 300–700 K because silicon-based thermoelectric devices are mainly used in this temperature range. The temperature dependence of the Seebeck coefficients of Si with different carrier densities is shown in Fig. 6a–b. The absolute values of *S*_total_ at low carrier density (*n* = 1.6 × 10^17^ cm^−3^) were higher than the corresponding *S*_total_ at high carrier density (*n* = 7.0 × 10^18^ cm^−3^), and *S*_total_ decreased more remarkably with increasing temperature at low carrier density. However, the absolute value of *S*_diff_ at the two carrier densities increased slightly with the temperature, whereas the absolute value of *S*_diff_ at low carrier density was higher than that at high carrier density. As a result, at low carrier density, the absolute value of *S*_pd_ decreased from − 636 to − 150 μV K^−1^ when the temperature was increased from 300 to 700 K. On the other hand, at high carrier density, the absolute value of *S*_pd_ decreased from − 402 to − 130 μV K^−1^ when the temperature was increased from 300 to 700 K. To evaluate the contribution of the phonon-drag effect to the Seebeck coefficient, the ratio of *S*_pd_ to *S*_total_ (*S*_pd_/*S*_total_) was calculated, as shown in Fig. 6c. At 300 K, the values of *S*_pd_/*S*_total_ at low and high carrier densities were 51% and 59%, respectively. These results indicate that the phonon-drag effect was sufficiently pronounced even at 300 K and at a high carrier density in the heavily doped region. Similar results have been reported by Zhou et al.^16^. The ratio of *S*_pd_/*S*_total_ for the two types of Si decreased as the temperature increased. At 700 K, the values of *S*_pd_/*S*_total_ at low and high carrier densities were 17% and 22%, respectively. The relationship between the phonon MFP and frequency at different temperatures and carrier densities is provided in the Supplementary Information (Fig. S2). Therefore, the phonon-drag effect is enhanced at low temperatures and high carrier densities in the heavily doped region. Currently, the results do not indicate the maximum temperature and carrier density values, but these findings are significant for designing thermoelectric materials with enhanced performance derived from the phonon-drag effect.

In the next step, we plan to investigate the phonon-drag effect of bismuth-telluride thin films with different deposition methods^38–41^, which exhibit high thermoelectric performance near 300 K, and further increase the thermoelectric performance using the phonon-drag effect. The application of mechanical strain is a conventional method of tuning the electronic and phonon properties of nanostructures. In particular, a large strain will result in distinct deformation of heavily doped lattice structures, which also has a significant effect on the Seebeck coefficient and thermal conductivity^42–45^. Therefore, based on our previous studies^46–48^, we intend to combine doping techniques with strain application in thin films to further enhance the phonon-drag effect.

## Conclusions

In this study, we measured the phonon and electron MFPs of Si single crystals doped with different amounts of phosphorus to investigate the phonon-drag effect. The phonon MFP was measured using nanoindentation and spot-periodic heating radiation thermometry, and the electron MFP was obtained from the electrical conductivity of Si single crystals. Electron MFP is more dependent on the carrier density than phonon MFP because electrons are charged particles and are therefore affected by the Coulomb potentials derived from impurities. The *S*_diff_ values derived from the electron transport properties are in good agreement with those in previous reports. However, the *S*_pd_ values derived from the phonon transport properties are very low. This phenomenon suggests that phonons with a normal mean free path (MFP) do not increase the Seebeck coefficient, but phonons with longer MFP and lower frequency increase the Seebeck coefficient via the phonon-drag effect. Therefore, to improve the thermoelectric performance, the carrier density should be optimized to effectively create a phonon-drag effect while reducing the thermal conductivity. Analysis of the temperature dependence indicated that the phonon-drag effect was enhanced at low temperatures and high carrier density in the heavily doped region. Even though the optimal temperature and carrier density for achieving the maximum thermoelectric performance have not been determined, the results of this study are significant for designing thermoelectric materials with enhanced performance derived from the phonon-drag effect.

## Methods

In this study, we used four types of phosphorus-doped n-type [111]-oriented Si single crystals with different doping densities and undoped [111]-oriented Si single crystals. The sample size was 20 mm × 20 mm, with a thickness of approximately 0.6 mm. Initially, the Si single crystals were immersed in a hydrofluoric acid solution diluted 50-fold with ion-exchange water for 1 min to clean the Si single crystals and then dipped in ion-exchange water for 1 min. After washing, the Si single crystals were air-dried.

The elastic modulus of the Si single crystals was measured at 300 K using an iMicro nanoindentation testing system (KLA Corporation) equipped with InForce1000 actuator heads and a finely pointed stylus of natural diamond (Berkovich-type) with an accuracy of ± 2%^49^. The maximum load varied between 100 and 500 mN, and the hold time at the maximum load was maintained at 1 s.

The electrical conductivities and Seebeck coefficients of the single Si crystals were measured at 300 K using a thermoelectric material evaluation system with an accuracy of ± 2% (OZMA-1; Ozawa Science). The thermal conductivity *κ* is defined as *κ* = *αρC*, where *α* is the thermal diffusivity, *ρ* is the density, and *C* is the specific heat. The thermal diffusivity was measured using a thermophysical property-measuring instrument (Thermowave Analyzer, Bethel) based on spot-periodic heating radiation thermometry at a heating frequency of 5–550 Hz with an accuracy of ± 3%^50^. Prior to the measurement, the front and back surfaces of the samples were coated with glassy carbon by spraying (blackening treatment) for efficient photothermal conversion and laser heat radiation. The frequency response of the phase delay was measured against the direction of the sample thickness using a frequency modulation method. The thermal diffusivity was determined using the correlation between the frequency and phase delay. The specific heat was utilized from the thermophysical properties database given in the literature (714 [J/(kg K)^−1^] at 300 K).

### Supplementary Information

Supplementary Figures.

## Data Availability

The authors declare that most data supporting the findings of this study are available in this paper and its Supplementary Information files. The remaining data generated and/or analyzed during the current study are available from the corresponding author upon reasonable request.
