# Supplementary material for: Determination of Seebeck coefficient originating from phonon-drag effect using Si single crystals at different carrier densities
Source: Sci Rep. 2023 Aug 18;13:13463. doi: 10.1038/s41598-023-40685-6 (PMC10439221; doi:10.1038/s41598-023-40685-6)
Supplement: Supplementary file 1 — Supplementary Figures. [file 41598_2023_40685_MOESM1_ESM.docx]

**Electronic Supplementary Information**

for

**Determination of Seebeck coefficient originating from phonon-drag effect using Si single crystals at different carrier densities**

Masataka Hase^1^, Daiki Tanisawa^1^, Kaito Kohashi^1^, Raichi Kamemura^2^, Shugo Miyake^2^, Masayuki Takashiri^1,a)^

^1^*Department of Materials Science, Tokai University, 4–1–1 Kitakaname, Hiratsuka, Kanagawa 259–1292, Japan*

^2^*Department of Mechanical Engineering, Kobe City College of Technology, Kobe, Hyogo 651–2194, Japan*

Corresponding Author

Masayuki Takashiri: takashiri@tokai-u.jp


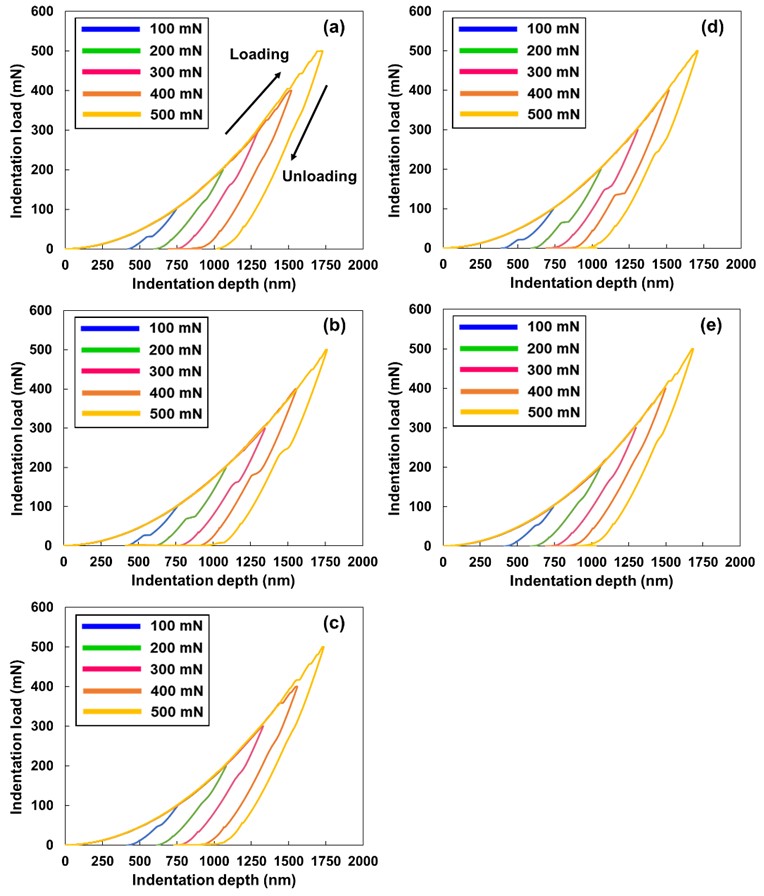


Figure S1. Load-displacement curves measured by nanoindentation at different carrier concentrations of Si single crystals. (a) 1.8×10^11^ cm^-3^, (b) 2.3×10^14^ cm^-3^, (c) 6.6×10^15^ cm^-3^, (d) 1.6×10^17^ cm^-3^, (e) 7.0×10^18^ cm^-3^.


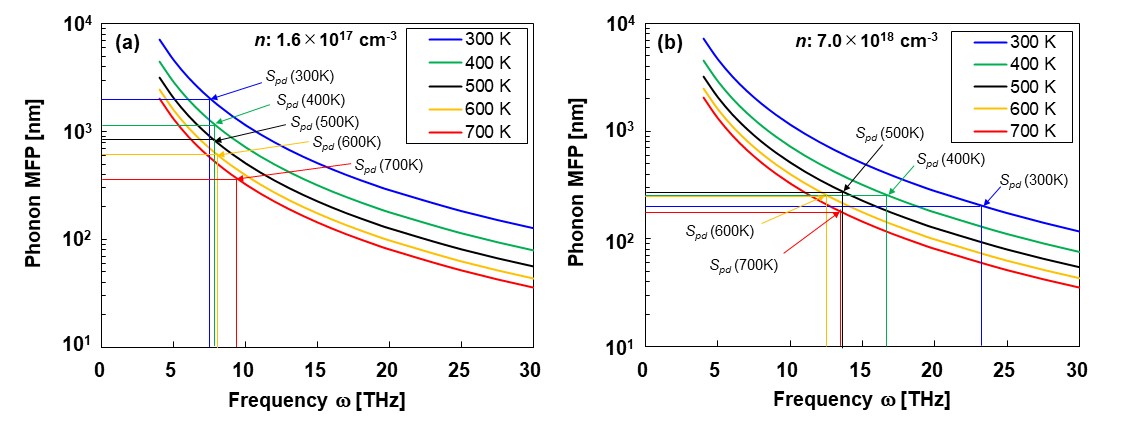
 The calculations were performed using the methods described in the main article. At a carrier density of 1.6 × 10^17^ cm^-3^, increasing the temperature led to a decrease in the phonon MFP, whereas the frequency increased. The change in the phonon MFP was more pronounced than the change in the phonon frequency. On the other hand, at a carrier density of 7.0 × 10^18^ cm^-3^, the relationship between the phonon MFP and frequency was generally the same as that at 1.6 × 10^17^ cm^-3^, but the phonon frequency changed to a greater extent than the phonon MFP.

Figure S2. Variation of phonon MFP and frequency with temperature and carrier density: (a) 1.6 × 10^17^ cm^-3^ and (b) 7.0 × 10^18^ cm^-3^.
